# Emotion in Motion: Weight Bias Internalization, Exercise Avoidance, and Fitness-Related Self-Conscious Emotions

**DOI:** 10.3390/healthcare12100955

**Published:** 2024-05-07

**Authors:** Sophie S. Smith, Gill A. Ten Hoor, Niharika Lakhote, Karlijn Massar

**Affiliations:** Department of Work & Social Psychology, Faculty of Psychology & Neuroscience, Maastricht University, P.O. Box 616, 6200 MD Maastricht, The Netherlands; ss.smith@student.maastrichtuniversity.nl (S.S.S.); gill.tenhoor@maastrichtuniversity.nl (G.A.T.H.); niharikalakhote@gmail.com (N.L.)

**Keywords:** weight bias internalization, self-conscious emotions, exercise avoidance, shame, guilt, overweight

## Abstract

Weight bias internalization (WBI), the process of internalizing negative attitudes and stereotypes towards overweight individuals, significantly impacts self-worth and health behaviors, such as exercise avoidance. In the current study, we focused on the mediating role of fitness-related self-conscious emotions, particularly shame and guilt. A cross-sectional study involving 150 self-described overweight Dutch women (age M = 49.63 ± 10.72) was conducted online. Participants completed measures assessing weight bias internalization, exercise avoidance, and body/fitness-related self-conscious emotions. Data were analyzed using linear regression and mediation analysis, controlling for age, BMI, and exercise frequency. The results show that weight bias internalization, guilt-free shame, and shame-free guilt uniquely predict exercise avoidance. Guilt-free shame partially mediated the relationship between weight bias internalization and exercise avoidance, indicating that increased internalized weight bias led to higher levels of guilt-free shame, which in turn contributed to exercise avoidance. Shame-free guilt did not act as a unique mediator. These findings underscore the importance of addressing weight bias internalization and fitness-related self-conscious emotions, particularly guilt-free shame, in interventions targeting exercise avoidance among overweight individuals. Strategies promoting self-compassion and reducing shame may prove beneficial in improving exercise behaviors and overall well-being.

## 1. Introduction

Weight-related bias is pervasive in modern (Western) society and includes negative attitudes about and stereotypes of individuals who are overweight, unjustly characterizing them as being lazy, unintelligent, lacking in self-control, and less competent [1]. Experiencing weight bias can have severe negative consequences for overweight or obese individuals and has been associated with overt unfair treatment in employment, healthcare, and education (i.e., discrimination), as well as social devaluation and denigration in interpersonal relationships and the media (i.e., stigmatization) [2]. The psychological and behavioral consequences of experiencing weight bias and weight stigmatization range from medication non-adherence and substance abuse to decreased mental health and social support [3].

Importantly, individuals may begin to internalize these biases and form negative attitudes towards themselves based on their weight. Weight bias internalization (WBI) refers to the process of being aware of the negative stereotypes and attitudes associated with being overweight, agreeing with these stereotypes, and applying them to oneself [4,5]. Weight bias internalization leads to a devaluation of self-worth and worse body image [6] and has been associated with worse physical, psychological, and behavioral health [7]. For example, greater weight bias internalization has been associated with a heightened cardiometabolic risk [8], an increase in weight cycling in women [9], higher levels of anxiety and depression, and reduced mental health-related quality of life [6]. Additionally, increased weight bias internalization is associated with disordered eating, including disinhibited eating, compensatory behaviors, and dietary restraint [7].

Being overweight or obese can lead to an avoidance of physical exercise and sports [10], and it has been suggested that particularly overweight individuals engage in such self-exclusion from sports or exercise due to weight bias internalization [11], but also as a coping strategy to avoid stigmatizing reactions from others [12]. This is problematic, given the evidence that engagement in and enjoyment of leisure-time physical exercise have positive effects on subjective well-being [13] and are associated with better mental health and lower levels of mental ill-health, notably depression [14]. In order to stimulate overweight individuals to engage in exercise, it is therefore crucial to know how weight bias internalization is related to exercise avoidance. Research has already established that weight bias internalization is associated with lower motivation for and enjoyment of exercise and physical activity [15,16], although this seems to be mainly restricted to social physical exercise or sports [12]. Recently, [17] showed that weight stigma, internalized weight stigma, and avoidance of physical exercise predicted psychological distress, signifying the importance of understanding the relationships between these constructs.

One theoretical perspective that offers insight into the mechanisms underlying the relationships between (internalized) weight stigma and engagement in physical exercise is self-determination theory (SDT) [18]. This theory states that increased intrinsic motivation to engage in behaviors, including exercise, may be expected when an individual feels the behavior is the result of willingness and choice, rather than the result of internal or external pressures. In other words, the basic psychological need for autonomy must be met in order for a certain behavior (e.g., physical exercise) to be intrinsically motivated and, as a result, be enjoyed and sustainably performed [17,18]. In the context of physical exercise and internalized weight stigma, external pressures may also consist of the awareness and internalization of an ideal or idealized body weight or shape. According to SDT, knowledge of such societal expectations will cause individuals to perceive a lack of autonomy in decisions to engage in physical exercise, which will decrease their intrinsic motivation and ultimately cause early quitting or avoidance of the desired behavior. Indeed, previous research [19,20] has shown that individuals who experienced weight stigmatization also reported a thwarting of their psychological need for autonomy, which in turn reduced their engagement in physical exercise.

Variables that could explain the relationship between weight bias internalization and exercise avoidance (i.e., mediating variables) have not been studied extensively yet. Feig et al. [21] identified exercise self-efficacy and depression as mediators, whereas Pearl and Dovidio [22] suggested belief in a just world might explain how weight bias affects exercise avoidance. Motivational variables, such as self-control—the ability to exert control over one’s behaviors through the effortful inhibition of unhealthy behaviors or the active development of healthy habits—have also been listed as possible mediating variables [23]. For example, [24] confirms the finding that internalized weight stigma is associated with an avoidance of physical exercise and adds the novel finding that weight stigma affects individuals’ capacity to exert self-control over weight management, causing lower engagement in physical exercise. Thus, although some explanatory variables in the relationship between internalized weight stigma and exercise avoidance have been identified, in the current study, we aim to add to the literature by focusing on the role of self-conscious emotions as possible mediators.

Self-conscious emotions—shame, guilt, and pride—are emotions that are generated in response to events that positively or negatively reflect on and influence one’s self-worth, and they are pivotal in motivating and influencing an individual’s thoughts, feelings, and behaviors [25]. Briefly, feelings of shame arise when an individual fails to meet their own or society’s expectations and cause avoidance and withdrawal, guilt emerges from the regret of (not) performing a certain behavior and often leads to reparative actions, and pride emerges as a result of having achieved a (socially) valued goal and promotes continuation of the actions causing this achievement [25]. Thus, even though shame and guilt both cause individuals to attribute the cause of an event to some internal factor, the two emotions differ with respect to the controllability of these internal characteristics: “[…] shame involves negative feelings about individual characteristics that are uncontrollable (e.g., the individual perceiving that they are not fit), whereas guilt involves negative feelings caused by a controllable factor such as an individual’s behavior (e.g., the individual perceiving that they are not doing enough to improve their own fitness)” [26]. In line with SDT, shame and guilt may arise due to individuals’ perception of a failure to meet societal standards for body weight and body shape, which in turn decreases (self-determined, autonomous) motivation for exercise [27].

In recent years, self-conscious emotions have been contextualized to the body and to exercise/fitness [26,27,28,29,30]. Nesbitt et al. [31] show that, compared to global shame and guilt, body-related shame and guilt were more strongly related to negative mental health outcomes such as depression, anxiety, and eating pathology. Furthermore, the associations between body-related or fitness-related shame, guilt, and pride and exercise behavior can be both positive and negative [26,28]. In both cases, these emotions can instigate behaviors that are socially valued, such as engaging in physical exercise (to alleviate feelings of guilt due to being inactive) or refraining from physical exercise (out of shame due to being overweight). However, different self-conscious emotions are associated with different motivations to exercise and instigate different behaviors. Indeed, shame and guilt have been associated with exercising for appearance-related reasons, whereas pride has been associated with exercise for health-related reasons [32]. Generally, however, the association between body-related shame and physical exercise is negative [33], whereas guilt has been found to have both positive [34] and negative associations with exercise [35]. A recent study by Sick et al. [34] established a causal relationship between daily fluctuations in feelings of guilt (but not shame) and increased subsequent moderate-to-vigorous exercise, suggestive of maladaptive coping to reduce a certain emotional state.

The literature review above thus indicates that a risk of exercise avoidance is particularly present among overweight individuals who have internalized societal weight-related stigmatizing stereotypes and attitudes. Moreover, there is evidence that fitness- and body-related self-conscious emotions negatively influence exercise. Insight into these processes may also provide starting points for possible interventions aimed at increasing the level of physical exercise for overweight individuals. The aim of the current study is to examine the relationship between weight bias internalization and avoidance of physical activity while considering the mediation of fitness-related self-conscious emotions—specifically, shame, guilt, and pride—among a sample of overweight women.

## 2. Materials and Methods

### 2.1. Participants and Procedure

The participants in the present study are a subset of a sample acquired for a project conducted in 40 nations [36]. A total of 307 Dutch female participants (age range: 16–65 years) completed an online survey via a Dutch research panel and were remunerated for their participation by the panel. Care was taken to draw a sample that was representative of the Dutch female population in terms of educational background, income, and age.

Here, we report on the subset of N = 150 women (mean age = 49.63, SD = 10.72) who indicated being overweight (see Section 2.2 for the filter question). Post hoc power analysis utilizing G*Power 3.1 [37], assuming a small-to-medium effect size (f = 0.08) with 3 predictors, reveals a power of 0.83 was obtained with this sample size, which is acceptable.

Participants received information about the study’s purpose and provided informed consent before commencing to answer the questions. All materials and procedures were approved by the Ethics Research Committee of Psychology and Neuroscience at Maastricht University (code 04_09_2012_S27).

### 2.2. Measures

In addition to their income, highest educational attainment (both categorized), age, and exercise frequency (categorized), participants’ height and weight were self-reported and were used to calculate BMI (range 19.61–55.56). For an overview of the sample characteristics, see Table 1 (please note: income and educational attainment were used only for sample description and will not be included in any analyses). Before participants completed the other measures, we asked them whether they were overweight (yes/no) and included a link to the Dutch Nutrition Agency website with information about healthy/unhealthy BMI ranges and where they could compute their own BMI if they were unsure whether they were overweight or not (see https://www.voedingscentrum.nl/nl/bmi-meter, accessed on 14 April 2024 ). Upon answering ‘Yes, I am overweight’, participants were directed to the measures listed below.

The WBIS [5] assesses the internalization of weight bias among overweight and obese individuals. It measures the belief in social stereotypes attributed to obesity and the negative self-evaluations because of one’s own weight. Participants were asked to indicate their level of agreement with eleven items on a seven-point Likert scale (1 = strongly disagree, 7 = strongly agree). Examples are “I feel uncomfortable with being overweight because of what other people may think of me” and “My weight determines my value as a person”. The internal reliability was good and in line with previous studies utilizing the WBIS, with [38] reporting Cronbach’s α = 0.87 and [39] α = 0.91. In the current research, α = 0.91.

We measured exercise avoidance on a 7-point scale (1 = strongly disagree, 7 = strongly agree) using three statements taken from [40] that were preceded by the statement ‘With respect to physical exercise…’: (a) I feel like other people are constantly judging me, (b) I’m uncomfortable with the idea of going to the gym where there are a lot of mirrors, (c) I don’t go to the gym because there are a lot of slim people there. In the current research, Cronbach’s α = 0.83, which is in line with reliability reports by [11], who reported a reliability of 0.79 for the full scale, and [41], who reported an α of 0.88.

The BSE-FIT [25] is a 16-item self-report measure assessing positive and negative emotional experiences of shame, guilt, and pride in a fitness/exercise context. For the current study, we measured only shame and guilt (4 statements each). On a 5-point scale, participants indicated how often they experience these emotions about their body in a fitness/exercise context. Example items are “I have felt ashamed about what my body can do physically” and “I felt guilty that I’m not doing enough to improve my fitness”. Castonguay [42] reported that for shame, Cronbach’s α = 0.84 and for guilt, α = 0.91, whereas [43] reported that for shame, α = 0.86 and for guilt, α = 0.90. In the current study, for shame, Cronbach’s α = 0.86, and for guilt, α = 0.91.

### 2.3. Data Analysis

SPSS v28 was used to analyze all data, including descriptives, correlations, and linear regression. We utilized the PROCESS v4.2 macro for mediation analyses (model 6) [44]. Since shame and guilt conceptually overlap and we aimed to investigate the unique contributions of each of these self-conscious emotions to the relationship between weight bias internalization and exercise avoidance, we utilized shame-free guilt and guilt-free shame. Shame-free guilt was assessed as the standardized residual associated with predicting guilt from shame, whereas guilt-free shame was the standardized residual associated with predicting shame from guilt, both obtained from linear regressions.

## 3. Results

Based on the correlations between the main variables (controlled for age, BMI, and exercise frequency; see Table 2), we decided to exclude pride from further analyses since this variable was not significantly associated with either weight bias internalization or exercise avoidance.

Next, a linear regression analysis was conducted, including BMI, age, and exercise frequency in Model 1, weight bias internalization in Model 2, and guilt-free shame and shame-free guilt in Model 3 (see Table 3). This analysis revealed that in the final model, the unique predictors were weight bias internalization (B = 0.70, t(142) = 6.74, *p* < 0.001), guilt-free shame (B = 0.53, t(142) = 2.62, *p* = 0.01), and shame-free guilt (B = 0.40, t(142) = 2.12, *p* = 0.04).

Then, we added guilt-free shame and shame-free guilt as mediators to the model (PROCESS model 6). These analyses revealed that guilt-free shame partially mediated the effect of weight bias internalization on exercise avoidance (total indirect effect β = 0.18, 95% CI [0.02, 0.36]; *R^2^* = 0.51, *F*(3, 145) = 50.74, *p* < 0.001), such that the total effect (β = 0.90, *t*(142) = 11.63, *p* < 0.001) was reduced when the indirect effect was added (direct effect β = 0.72, *t*(142) = 6.93, *p* < 0.001). Guilt-free shame uniquely explained part of the effect of weight bias internalization, but shame-free guilt or both mediators together were not unique mediators (see Table 3 for details).

## 4. Discussion

Weight bias internalization can have serious negative consequences for health and health behaviors, as well as mental health [17]. There is also increasing evidence that internalized weight stigma affects enjoyment of physical exercise and may cause disengagement from physical exercise [10,11]. Even though some explanatory variables have been identified in previous research [21,24], more knowledge is needed about the relationship between internalized weight bias and exercise avoidance. Therefore, in the current study, we focused on the role of fitness-related self-conscious emotions since specifically shame and guilt have previously been shown to predict lower engagement in physical exercise [32]. Our results show that in a sample of women who self-described as overweight, exercise avoidance was uniquely predicted by weight bias internalization, guilt-free shame, and shame-free guilt. Furthermore, we show that only guilt-free shame partially mediated the relationship between weight bias internalization and exercise avoidance, whereas shame-free guilt and both emotions together did not.

Given that shame and guilt share common variance, we utilized shame-free guilt and guilt-free shame to be able to study their unique associations. The finding that only guilt-free shame acts as a mediator can be explained with research that consistently indicates that shame is associated with feeling inferior, worthless, and unattractive—both in the eyes of others and of oneself—and causes withdrawal and avoidance behaviors [23,45]. Similarly, internalized weight bias causes exercise avoidance and is strongly associated with body-related shame [46]. In the current study, we link these findings and report that overweight women who experienced increased internalized weight bias also reported increased shame, which in turn increased exercise avoidance. Such avoidance of physical exercise may enhance feelings of body-related shame, increasing the risk of a vicious cycle to emerge among women who already suffer from internalized sociocultural negative attitudes about body weight.

These findings are also in line with self-determination theory [18]: internalized weight stigma can be seen as the result of a stigmatizing and controlling social context—i.e., the Western European weight-centric society. SDT predicts that such social contexts will thwart individuals’ basic psychological needs, notably autonomy, due to both a subjective sense of failing to meet expectations and the experience of active rejection by others. Psychological need thwarting in turn causes psychological and behavioral maladjustments, including controlled motivation or amotivation, as well as lowered mental wellbeing and avoidant or defiant behaviors [46]. In the context of physical exercise, this perspective could thus explain why internalized weight stigma causes exercise avoidance. Further, SDT predicts that shame could act as a mediating variable since it is likely to be experienced as the result of a perceived failure to meet an ideal or idealized body shape and weight and might be considered the emotional expression of need frustration [47]. Indeed, Turner and colleagues [48] state that shame has “… devastating effects on ensuing motivation and related goal-striving behavior” (p. 80).

Interestingly, shame-free guilt did not act as a unique mediator. This could be due to the fact that guilt, in contrast to shame, is an emotion that instigates reparative actions [31] and as such may not form part of the path from internalized weight bias to exercise avoidance. Indeed, there are studies that show that a positive relationship between guilt and exercise may stem from maladaptive coping to reduce the emotion rather than an intrinsic motivation to increase one’s fitness [31]. In a similar vein, Lucibello et al. [26] report that, whereas shame mediates the relationship between a perception of being too heavy and increased screen time (i.e., sedentary behavior), this same pattern was not present when guilt was included as a mediator. Therefore, it could be the case that guilt does not explain (part of) the relationship between internalized weight bias and the absence of exercise—whether this means active avoidance or increased sedentary behavior.

### 4.1. Limitations

Although this is one of the first studies analyzing these variables together—i.e., the influence of fitness-related self-conscious emotions on the relationship between weight bias internalization and exercise avoidance—and, as such, adds to the literature, there are also some limitations that need to be acknowledged. First, due to its cross-sectional design, causality and changes over time cannot be established. Specifically, although theoretically we assume that shame and guilt are the result of weight bias internalization and cause exercise avoidance, as stated above, it is most likely that this relationship is reinforced through a negative cycle. Including additional variables, such as body satisfaction, exercise habits, and physical activity, could have been beneficial to develop a deeper understanding of how weight bias internalization and the associated self-conscious emotions influence an individual’s approach to fitness-related behaviors. This might shed more light on these associations. Second, our sample was limited to women who self-described as being overweight. Even though we verified their BMI, which for this group was M = 30.50, which is classified as ‘overweight’, and we took great care to utilize a representative sample from the general public in the Netherlands, utilizing self-reported overweight, height, and weight ultimately is not as accurate as objective measurements. Last, we excluded women who self-reported not being overweight, precluding the possibility of comparing that group to our current sample, which does not allow us to verify whether the pattern of results observed for overweight women might also occur for women who did not self-describe as being overweight. Future research should therefore focus on expanding the diversity and inclusivity of the research population by, e.g., including men and women with different ethnicities and gender identities in the sample. Research has found that weight bias internalization is associated with different outcomes in men and women, suggesting there is a need for more comprehensive studies focusing on men [9].

### 4.2. Implications and Suggestions for Further Research

Our results also have implications for practitioners and intervention development. Specifically, our results indicate that self-conscious emotions, in particular shame and, to a lesser extent, guilt, might be relevant change objectives for interventions aimed at reducing exercise avoidance among (self-described) individuals with overweight. Specifically, programs or interventions aimed at increasing self-compassion may be beneficial since self-compassion may protect against the negative affective consequences of body dissatisfaction and weight bias internalization. For example, higher levels of self-compassion over three years were associated with lower levels of body-related guilt, shame, embarrassment, and envy [49], and increased self-compassion improved body image, specifically among individuals with internalized weight bias [50]. Self-compassion interventions can take on many forms, ranging from writing exercises to group-based psychoeducation, and result in small-to-moderate positive effects (compared to control conditions; see [51]). They can often be self-administered and are cost-effective. Further, specific interventions aimed at reducing shame include cognitive behavioral therapy and mindfulness-based acceptance programs [52].

In future studies, in addition to adding a focus on men and utilizing a larger and more diverse sample, a more explicit inclusion of variables central to SDT [18] could be included. Specifically, a minitheory of SDT, basic psychological needs theory (BPNT) [53], might provide more insight into the relationships between internalized weight bias and psychological need frustration, as well as the ensuing emotional reactions and behavioral patterns. Similarly, we urge researchers to pay attention to self-control processes—including emotion regulation—as a possible explanation for the relationships between weight stigma, self-conscious emotions, and exercise avoidance [23]. Here, the consequences of (depleted) self-control should be taken into account, i.e., both the inhibition of maladaptive behaviors (e.g., increased binge eating) as well as effortful engagement in desired behaviors (e.g., physical exercise).

Our findings furthermore suggest that a broader perspective on opportunities for physical activity might offer new insights into the relationships between internalized weight stigma, self-conscious emotions, and exercise avoidance [54]. Thus, in addition to leisure time exercise and sports, researchers could take tourism, physical education, or physiotherapy into consideration as opportunities for physical activity for individuals with regular weight as well as overweight or obese individuals.

## 5. Conclusions

Our results show that exercise avoidance was uniquely predicted by weight bias internalization, guilt-free shame, and shame-free guilt, and that only guilt-free shame partly mediated the relationship between weight bias internalization and exercise avoidance. Our findings suggest that focusing on reducing negative self-conscious emotions in regard to one’s weight, specifically in alleviating shame, may be beneficial to decreasing exercise avoidance in overweight individuals.

## Figures and Tables

**Table 1:** Sample description (N = 150).

	Mean (SD)	% Sample
Age	49.63 (10.72)	
BMI	30.13 (5.37)	
Exercise frequency (week)		
Never/seldom		32.7
Sometimes		54.7
Often		12.7
Income level		
Below average *		34
Average		20
Above average		26
Unknown		20
Level of educational attainment		
High		27.3
Middle		57.3
Low		15.3

Note: * average Dutch income level (modal income) was provided in the question.

**Table 2:** Correlations between main variables and mean scores (SD).

	Mean (SD)	1	2	3	4
1. Weight bias internalization	3.34 (1.24)	-			
2. Exercise avoidance	3.15 (1.61)	0.69 **	-		
3. Shame	2.54 (0.84)	0.67 **	0.58 **	-	
4. Guilt	2.71 (0.91)	0.54 **	0.45 **	0.77 **	-
5. Pride	2.52 (0.84)	−0.02	−0.07	−0.09	−0.16

Note: Correlations controlled for age, BMI, and exercise frequency (N = 144); ** *p* < 0.001.

**Table 3:** Linear regression and mediation analyses predicting exercise avoidance (EA).

Linear Regression	B	SE	t	R^2^	F Change
Model 1				0.10	5.06 **
Age	−0.01	0.009	−1.51		
BMI	−0.004	0.02	−0.23		
Exercise frequency	−0.17	0.15	−1.18		
Model 2				0.52	127.27 **
WBIS	0.70 **	0.10	6.74		
Model 3				0.54	3.48 *
Guilt-free shame	0.53 *	0.20	2.62		
Shame-free guilt	0.40 *	0.19	2.12		
**Indirect effects**	B	Boot SE	Boot 95% CI		
WBIS → GF shame → EA	0.18	0.10	[0.02, 0.40]		
WBIS → SF guilt →EA	0.14	0.07	[−0.01, 0.29]		
WBIS → GF shame → SF guilt → EA	−0.13	0.08	[−0.31, 0.01]		

Note: WBIS = Weight Bias Internalization Scale; EA = exercise avoidance; CI = confidence interval. ** *p* < 0.01; * *p* < 0.05.

## Data Availability

Data are available upon request: email karlijn.massar@maastrichtuniversity.nl.
